# To the Country Practitioner

**Published:** 1877-08-20

**Authors:** 


To the Country Practitioner.—J. F. Mooty, M.D., of Louisiana, in a private communication complimenting the practical character of The Record, and the interest which it takes in the village and country doctor, thus concludes:

"I wish to say this to my brethren of the country: The Record presents the best opening for you I have yet seen. Hospital statistics and long prosy dissertations do not suit me. I wait on a live people—and I presume it is so with all of you. Syrup of morphia, beef-tea and brandy will not cure a robust and otherwise healthy young man of pneumonia in this country. There is a marked difference between city hospital and country practice. If you can get the country physicians interested sufficiently to send up "home-made reports,' ask questions, and procure answers through The Record, its present great usefulness will be still further increased and much practical benefit will accrue to its readers and the profession generally."

